# Diet and Nutrients Intakes during Infancy and Childhood in Relation to Early Puberty: A Systematic Review and Meta-Analysis

**DOI:** 10.3390/nu14235004

**Published:** 2022-11-24

**Authors:** Jingyi Tang, Peng Xue, Xiaoxia Huang, Cuilan Lin, Shijian Liu

**Affiliations:** 1 Sanya Women and Children’s Hospital, Managed by Shanghai Children’s Medical Center, School of Medicine, Shanghai Jiao Tong University, 339 Yingbin Road, Sanya 572022, China; 2 Department of Clinical Epidemiology and Biostatistics, Children Health Advocacy Institute, Managed by Shanghai Children’s Medical Center, School of Medicine, Shanghai Jiao Tong University, Shanghai 200127, China; 3 School of Public Health, School of Medicine, Shanghai Jiao Tong University, Shanghai 200127, China; 4 Health Care Center for Primary and Secondary Schools, Zhongshan 528403, China; 5 Boai Hospital of Zhongshan Affiliated to The Second School of Clinical Medicine, Southern Medical University, Zhongshan 528405, China

**Keywords:** diet, nutrients, early puberty, infancy, childhood

## Abstract

The onset of puberty has become earlier over the decades, and nutrients and diet are related to the timing of puberty onset. Hence, we aimed to investigate the association between diet or nutrients in infancy, childhood and early puberty. PubMed, Embase, and Cochrane library were searched systematically up to 15 April 2022. The pooled relative risks (RRs) or regression coefficients (beta) were estimated using the random-effect model or fixed-effect model according to the heterogeneity between studies. Twenty-two articles on diet or nutrients in childhood and six about breastfeeding in infancy were included. The prolonged breastfeeding duration in infancy could reduce the risk of early menarche (beta 0.31, 95% CI: 0.01, 0.60, *p* = 0.045). The high intake of yogurt was associated with a 35% reduction in the risk of earlier menarche (RR 0.65, 95% CI: 0.47, 0.89, *p* = 0.008). Girls with severe food insecurity experienced later menarche (RR 0.81, 95% CI: 0.67, 0.98, *p* = 0.027). Conversely, due to the high intake of protein, the risk of early menarche increased by 8% (RR 1.08, 95% CI: 1.01, 1.16, *p* = 0.016). High intake of yogurt, longer duration of breastfeeding, and food insecurity decreased the possibility of earlier menarche, while high intake of protein increased that risk. As a modifiable factor, diet and nutrients in infancy and childhood provide new insights into the future prevention of early puberty.

## 1. Introduction

Puberty is a pivotal period in the lifecycle. During this period, people experience the transformation from children to adults, attaining both somatic growth and reproductive maturity. For boys, genital growth, pubic hair development, and voice break are recognized as major puberty milestones. For girls, breast growth, pubic hair development, and menarche are the main secondary sexual characteristics in puberty [1]. There is a trend that the prevalence of early puberty is increasing worldwide, particularly in girls [2,3]. Early puberty increases many subsequent physical and psychological unhealthy conditions or diseases. Indeed, several studies showed a significant association between early puberty and endocrine diseases, for example, early menopause, breast cancer, and prostate cancer [4,5,6].

The timing of puberty is determined by numerous risk factors with their complex interplay. Genes partly determine when an individual’s puberty begins. For instance, makorin ring finger protein 3 (*MKRN3*) and delta-like noncanonical Notch ligand 1 (*DLK1*) are key genetic drivers in the progression of central precocious puberty [7,8]. Obesity also plays a critical role [9]. Ferrari et al. [10] indicated that girls with higher BMI had a higher risk of early menarche and breast development. In addition, an incomplete family structure may contribute to early puberty [11].

As a modifiable factor, the influence of diet and nutrients on the timing of puberty onset receives keen attention. However, the findings showed an inconsistent association. Rogers et al. [12] found that the high intake of meat predisposed girls to earlier menarche in British, while this positive association was not observed by Wu et al. [13]. Likewise, the influence of carbohydrate intake on early menarche was also controversial [14,15]. Several studies pointed out that the duration of breastfeeding was associated with the timing of menarche and breast development [16,17]. Moreover, increasing evidence indicates that food insecurity also affects the age of menarche [18]. The previous studies mainly focused on children’s nutritional status and subsequent menarche onset, leaving gaps in understanding other pubertal milestones and nutritional status in early life. Therefore, this systematic review and meta-analysis aimed to comprehensively clarify the influence of diet and nutrients in infancy and childhood on early puberty, providing new insights into formulating policies for the prevention of early pubertal development.

## 2. Methods

We performed this systematic review and meta-analysis following the Preferred Reporting Items for Systematic Reviews and Meta-Analyses (PRISMA) guideline. This study was registered with PROSPERO (CRD42022349856).

### 2.1. Search Strategy

We searched comprehensively in PubMed, Embase, and Cochrane library for relevant studies published from inception to 15 April 2022. We did not impose any language restrictions nor limit the research types. To ensure a thorough literature search, the following keywords were used, food, diet, breastfeeding, milk, meat, dairy, carbohydrate, fat, protein, food insecurity, early puberty, precocious puberty, premature, thelarche, and menarche. Boolean operators “OR” and “AND” were used to combine these search terms. To retrieve articles more accurately, we limited the age of the research subjects to less than 18 years old, as puberty generally ends at this age. We also reviewed references of included articles to identify relevant literature.

### 2.2. Inclusion and Exclusion Criteria

To be eligible, the published articles should meet the following criteria: (a) They were epidemiological studies, including cross-sectional, case-control, or cohort studies. (b) Different levels of diet or nutrient intake were considered as exposure. The way of assessment was food frequency questionnaires, food diaries, or dietary recall interviews. The outcome of interest was the number of children at different pubertal stages and/or the age at pubertal milestones. Pubertal indicators were obtained by clinical diagnosis, questionnaire, or self-reporting. (c) Studies investigated the association between early puberty and diet or nutrient intake. The adjusted odds ratio (OR), relative risk (RR), hazard ratio (HR), or regression coefficients (beta), as well as its 95% confidence interval (CI), were provided.

Studies were excluded if they (a) did not study specific diet or nutrients but focused on only dietary patterns; (b) selected subjects with medical conditions or diseases that would cause early puberty, such as adrenal dysfunction, pituitary tumor, thyroid hypofunction; (c) did not assess pubertal outcomes (breast, pubic hair, and testicular development) according to the Tanner staging; (d) were the only one article to show the relationship and could not be combined using meta-analysis.

### 2.3. Literatures Screening and Data Extraction

The studies obtained through the search strategy were screened to rule out duplicates. Then, the review of the title and abstract was performed. Among the full-text articles, ineligible articles were removed according to the exclusion criteria. For all included studies, the following characters were extracted, including first author, year of publication, country, study design, health status, age, gender, sample size, type of diet or nutrients, the pubertal outcome of interest, ORs, RRs, HRs or beta with 95% CIs for the relationship between diet or nutrients and early puberty. Confounding factors were taken into consideration.

### 2.4. Quality Assessment

The Newcastle–Ottawa Scale (NOS) was used to evaluate the quality of cohort and case-control studies. It assessed the risk of bias from three domains of selection, comparability, and outcome, giving scores ranging from 0 to 9. In this article, we defined the criterion controlling important confounding factors: maternal age at menarche, maternal education level, household income, weight, and height. Referring to the previous meta-analysis [19], the studies were deemed to be low, moderate, and high quality according to the score “0–3”, “4–6”, and “7–9”, respectively. The Agency for Healthcare Research and Quality (AHRQ) scale was used to assess cross-sectional studies. Scores of “0–3”, “4–7”, and “8–11” received through an 11-item checklist were considered as low, moderate, and high quality, respectively.

### 2.5. Statistical Analysis

The effect size measures used to report the relationship between diet or nutrients and puberty outcome were ORs, RRs, HRs, or beta with 95% CIs. We selected the risk estimates for the highest exposure group compared to the lowest or reference group with the adjusted model. Given that the relevant articles are scarcely available, the ORs and HRs were deemed equivalent to RRs and then pooled together directly [20]. The pooled risk estimates were depicted graphically with forest plots. Additionally, *I*^2^ and *p* statistical variables were used to evaluate the heterogeneity between studies. A random-effect model was used in the presence of significant heterogeneity (*I*^2^ > 50% or *p* < 0.1), otherwise, the fixed-effect model was selected. For the issues with more than three articles, sensitivity analysis was performed by omitting one study sequentially to assess the robustness of the results. Besides, Egger’s test was conducted to inspect the publication bias. Unfortunately, we were unable to conduct the subgroup analysis to explore possible sources of heterogeneity between studies owing to the limited number of studies. All statistical analyses were performed using Stata 16.0 (Stata Corp LLC, College Station, TX, USA).

## 3. Results

### 3.1. Literature Screening and Characteristics of Included Studies

**Figure 1:**
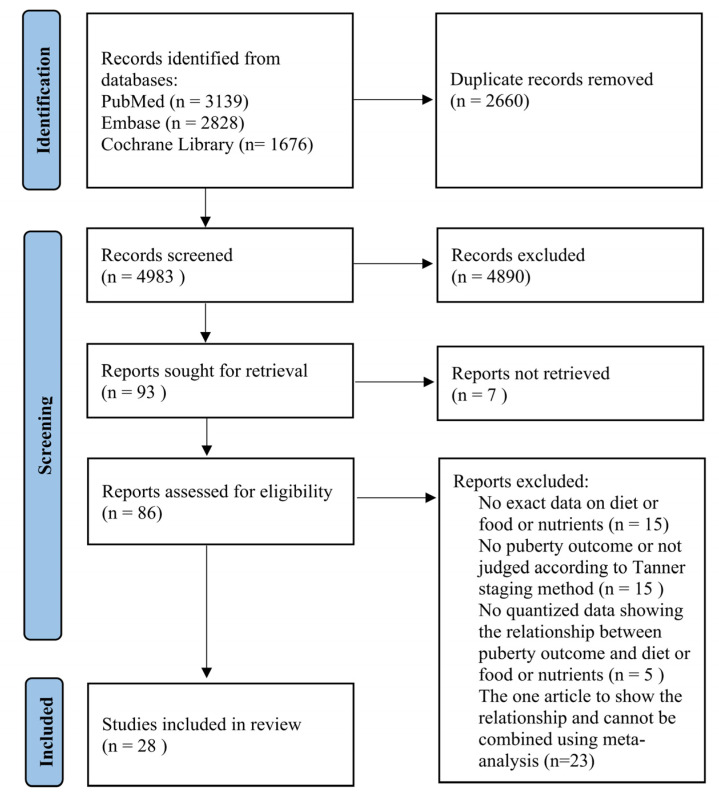
Flow chart for study selection in this systemic review and meta-analysis.

The process of the systematic search is shown in Figure 1. We obtained 7643 potentially eligible articles from PubMed, Embase and Cochrane Library, of which 2660 duplicates were removed. After further reviewing the title and abstract, 86 articles were eligible for full-text assessment. Owing to irrelevance to the inclusion criteria, 58 articles were ruled out, and the excluded reasons were presented in Supplementary Table S1. Finally, 28 articles were included in this systemic review and meta-analysis.

The characteristics of the 28 included articles are presented in Table 1. Briefly, 22 articles investigated the association between childhood diet or nutrients and early puberty. In addition, six articles [16,21,22,23,24,25] provided the effects of breastfeeding duration in infancy on the timing of menarche and breast development. Among the included studies, five were cross-sectional one was a case-control study [26], and 22 were cohort studies. These studies were performed in different countries around the world with a large sample of 162,073. Studies evaluating the risk of childhood diet and nutrients primarily used hazard ratios, providing benefits to test the causal relationship.

### 3.2. Quality Assessment

The details of quality assessment based on NOS are shown in Supplementary Table S2. The studies evaluated from three domains of selection, comparability, and outcome were all moderate or high quality. The self-report of menarche was the main cause of the quality decline. Age at menarche could not be obtained by the clinical examination like other pubertal milestones, such as the Tanner stage of breast development. In addition, the inadequacy of cohort follow-up and lack of adjustments for confounding factors would lower the scores. Similarly, six studies evaluated by AHRQ were graded as moderate or high quality.

### 3.3. Findings from the Studies

#### 3.3.1. Association of Breastfeeding Duration and Early Puberty

**Figure 2:**
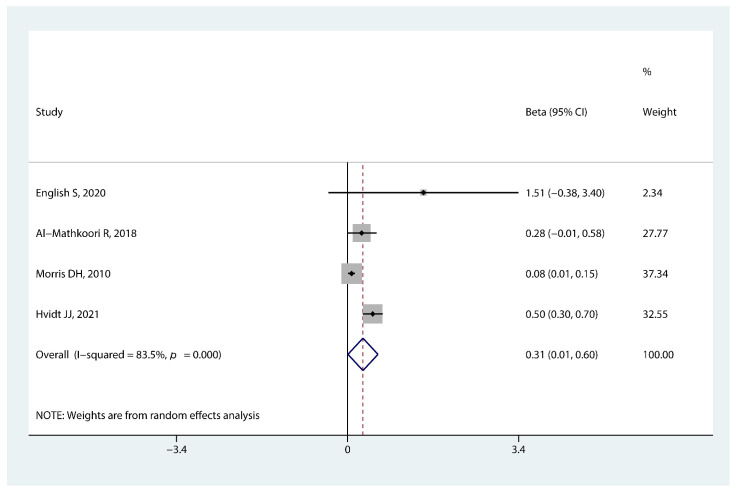
Forest plot of the association between breastfeeding duration in infancy and early menarche onset [21,22,23,24].

As illustrated in Figure 2, there was a significant association between breastfeeding duration in infancy and timing of menarche (beta 0.31, 95% CI: 0.01, 0.60, *p* = 0.045). The combined beta indicated 0.31 months later timing of menarche onset for every additional month of breastfeeding duration in girls.

#### 3.3.2. Association of Total Energy and Macronutrients Intake and Early Puberty

**Figure 3:**
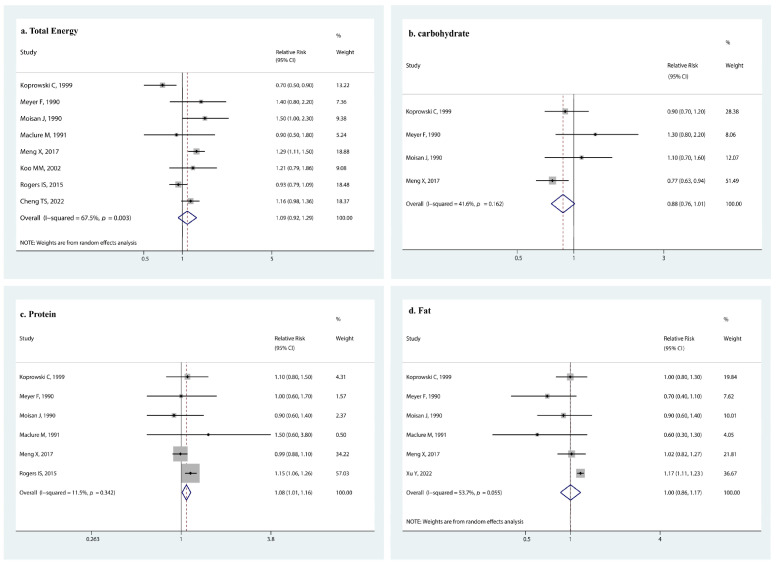
Forest plot of relative risk of early menarche onset for (**a**) total energy intake, (**b**) carbohydrate intake, (**c**) protein intake, and (**d**) fat intake [12,14,15,26,35,36,37,38,39].

We did not observe an association between early menarche and total energy intake (RR 1.09, 95% CI: 0.92, 1.29, *p* = 0.296, Figure 3a), carbohydrate intake (RR 0.88, 95% CI: 0.76, 1.02, *p* = 0.072, Figure 3b), and fat intake (RR 1.00, 95% CI: 0.86, 1.17, *p* = 0.965, Figure 3d). However, protein intake increased the risk of early menarche onset by 8% (RR 1.08, 95% CI: 1.01, 1.16, *p* = 0.016, Figure 3c).

#### 3.3.3. Association of Micronutrients and Phytochemicals Intake and Early Puberty

**Figure 4:**
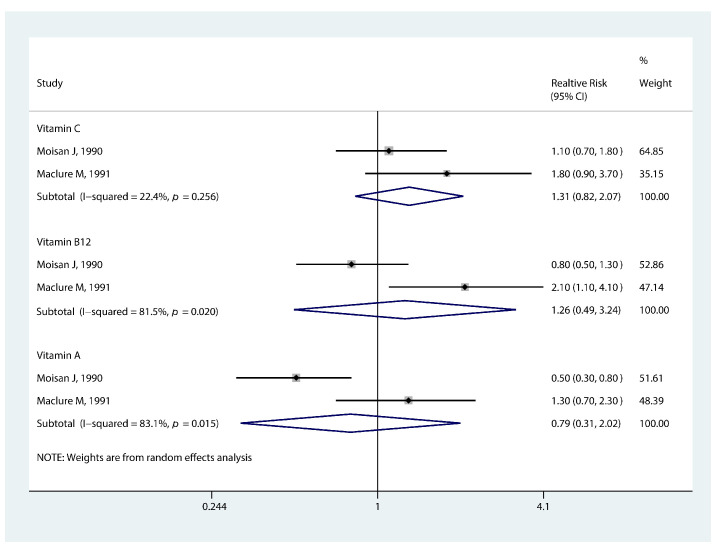
Forest plot of relative risk of early menarche onset for vitamins [26,35].

The pooled RR for the association between vitamins and earlier menarche onset is shown in Figure 4. The highest vitamin C intake was not significantly linked to earlier menarche compared to the lowest level (RR 1.31, 95% CI: 0.82, 2.07, *p* = 0.254). A similar relationship was observed for vitamin A (RR 0.79, 95% CI: 0.31, 2.02, *p* = 0.629) and vitamin B12 (RR 1.26, 95% CI: 0.49, 3.24, *p* = 0.630). Besides, Wolff et al. [43] and Mervish et al. [44] found lignan intake was irrelevant to the risk of earlier breast growth and pubic hair development; flavonols intake was not related to the development of pubic hair. While, Mervish et al. [44] discovered a relationship between breast growth and flavonols intake (HR 0.74, 95% CI: 0.61, 0.91).

#### 3.3.4. Association of Milk, Dairy, Yogurt Intake, and Early Puberty

**Figure 5:**
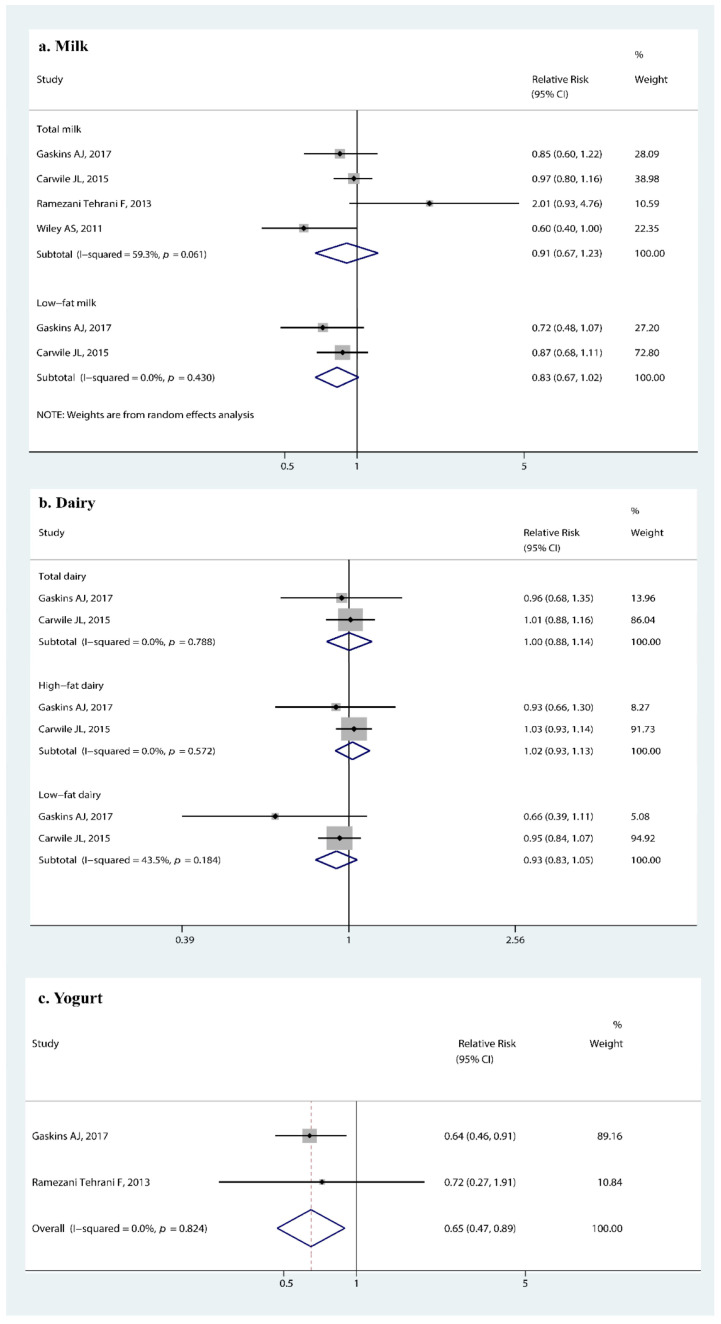
Forest plot of relative risk of early menarche onset for (**a**) milk intake, (**b**) dairy intake, and (**c**) yogurt intake [27,31,32,34].

We found that yogurt may reduce the risk of early menarche (RR 0.65, 95% CI: 0.47, 0.89, *p* = 0.008, Figure 5c). The pooled RR corresponded to an approximately 35% reduction in the risk of earlier menarche for girls. We did not observe a significant association between earlier menarche and milk (RR 0.91, 95% CI: 0.67, 1.23, *p* = 0.527, Figure 5a) or dairy intake (RR 1.00, 95% CI: 0.88, 1.14, *p* = 0.965, Figure 5b).

#### 3.3.5. Association of Meat Intake and Early Puberty

**Figure 6:**
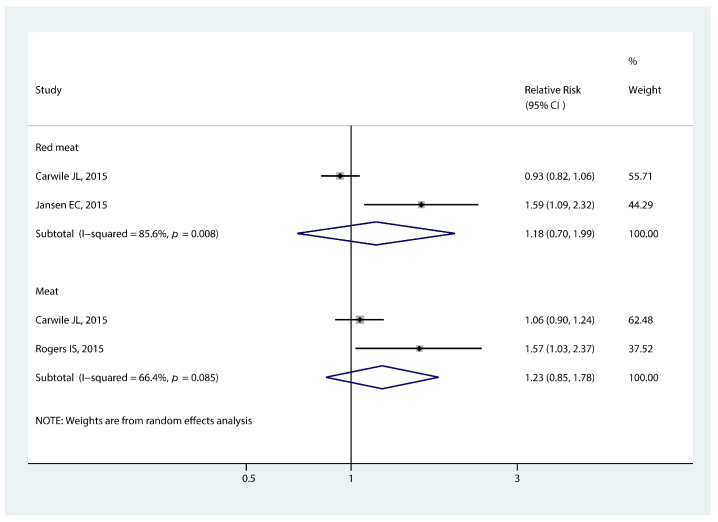
Forest plot of relative risk of early menarche onset for meat and red meat [12,30,31].

We did not observe a significant association between meat intake and earlier menarche (RR 1.23, 95% CI: 0.85, 1.78, *p* = 0.280, Figure 6). There was a similar relationship between red meat intake and the timing of menarche (RR 1.18, 95% CI: 0.70, 1.99, *p* = 0.536, Figure 6).

#### 3.3.6. Association of Soy Intake and Early Puberty

**Figure 7:**
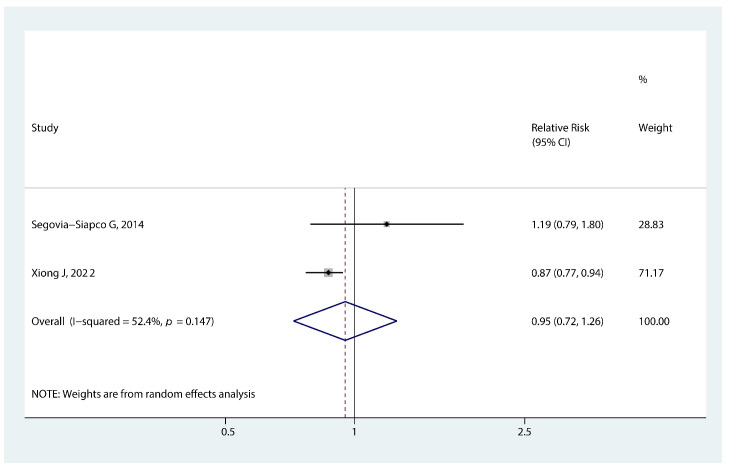
Forest plot of relative risk of early menarche onset for soy intake [41,42].

As shown in Figure 7, the pooled RR showed a nonsignificant association between soy intake and earlier menarche (RR 0.95, 95% CI: 0.72, 1.26, *p* = 0.730).

#### 3.3.7. Association of Food Insecurity and Early Puberty

**Figure 8:**
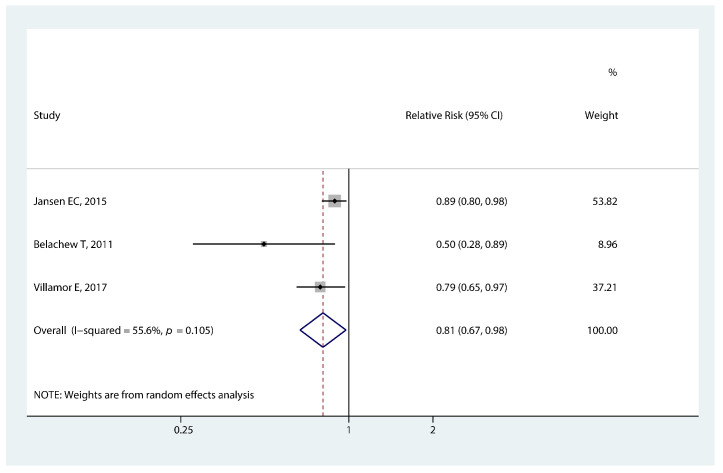
Forest plot of relative risk of early menarche onset for food insecurity [30,33,40].

The significant association between food insecurity in developing countries and the timing of menarche is presented in Figure 8. Girls who experienced food insecurity in childhood had a lower risk of early menarche (RR 0.81, 95% CI: 0.67, 0.98, *p* = 0.027).

### 3.4. Sensitivity Analysis and Publication Bias

The sensitivity analysis confirmed the robust association between the delay of age at menarche and prolonged breastfeeding duration or food insecurity (Supplementary Figures S1 and S2). However, the exclusion of the Rogers et al. study [12] altered the association between protein intake and earlier menarche to insignificant (RR 1.00, 95% CI: 0.91 to 1.11, Supplementary Figure S3c). Conversely, omitting Koprowski et al. study [15] materially changed the association between total energy intake and earlier menarche (RR 1.16, 95% CI: 1.01, 1.33, Supplementary Figure S3a). Likewise, the association between carbohydrate intake and earlier menarche became significant after removing Meyer et al. study [36] (RR 0.85, 95% CI: 0.73, 0.98, Supplementary Figure S3b) or Moisan et al. study [26] (RR 0.85, 95% CI: 0.73, 0.99, Supplementary Figure S3b). Publication bias was only detected for the studies of carbohydrate (Egger’s test: *p* = 0.006) and fat intake (Egger’s test: *p* = 0.000).

## 4. Discussion

In this systematic review and meta-analysis, our findings suggested that prolonged breastfeeding duration and high intake of yogurt had protective effects on earlier menarche. In addition, we observed a significant association between food insecurity and delayed menarche onset. In contrast, girls with a high intake of protein were more likely to experience earlier menarche. However, we did not observe significant associations of early puberty with total energy intake, carbohydrate, fat, milk, dairy, meat, vitamin, lignan, flavonols, and soy intake.

### 4.1. Prolonged Breastfeeding Duration and Delayed Puberty

The present meta-analysis suggested that delayed timing of menarche is related to breastfeeding duration early in life. Studies have also pointed out a preventive effect of prolonged breastfeeding duration on earlier breast development [16,25]. Insulin-like growth factor-1 (IGF1) may be of major importance in this association, as it can promote pubertal development by alleviating the inhibitory effect of dynorphin (*DYN*) on gonadotropin-releasing hormone (GnRH) [45]. The evidence is that the protein content of formula milk is much higher than human milk, resulting in a higher level of IGF1 [8,46]. Moreover, the breastfed infants presented a subsequent lower risk of obesity or overweight after four months postnatal [47]. Studies focusing on early-life growth discovered a negative correlation between weight in late infancy and the timing of menarche [48,49]. Furthermore, the early adiposity rebound (EAR) facilitates understanding this association. Adiposity rebound occurring before five years old is an established risk factor for downstream obesity [50]. Children who breastfed for more than four months were less likely to experience the EAR, reported in a large longitudinal study [51]. The risk of early puberty caused by obesity is, therefore, lower in children avoiding experiencing EAR. Hence, breastfeeding for at least six months, as recommended by the latest Dietary Guidelines for Americans [52], is feasible and imperative.

### 4.2. High Yogurt Intake and Delayed Puberty

Girls with high yogurt intake might have a lower likelihood of earlier menarche onset. Obesity is a recognized cause of early puberty, and several studies have focused on yogurt protein and anti-obesity, with mixed findings [53,54]. Meanwhile, insulin resistance, via its obesity-inducing impact, has a vital impact on advancing menarche onset [55]. According to a national cohort study in the USA, children who had yogurt regularly had a healthier insulin profile [56], but this impact might be influenced by the amount of fat in yogurts [57]. Additionally, preliminary studies suggested that probiotic bacteria in yogurt might prevent obesity by altering the gut microbiome [58,59].

### 4.3. Food Insecurity and Delayed Puberty

As defined, food insecurity is limited or uncertain availability of nutritionally adequate and safe foods or limited or uncertain ability to acquire acceptable foods in socially acceptable ways [60]. In underdeveloped nations and areas, food insecurity mainly refers to hunger and undernutrition, which has become a prevalent worry [61,62]. Infants and children experiencing food insecurity are prone to thinness and stunting [63,64]. Girls who are stunted or thin have later menarche onset in developing countries [65,66]. Taken together, these findings support the possible causal relationship between food insecurity and delayed menarche that we discovered. Noteworthy, this connection was denied by a cross-sectional study conducted in the US [29]. This discrepancy might be ascribed to the fact that in affluent countries, food insecurity is more related to unhealthy food like fried food and sugared drinks.

### 4.4. High Protein Intake and Earlier Puberty

High protein intake during childhood might be an effective predictor of early menarche. As previously stated, insulin resistance is related to earlier puberty onset. The long-term consumption of high-protein diets brings a higher risk of insulin resistance [67]. With this background, it can be hypothesized that insulin resistance is an intermediary factor linking high protein intake to earlier menarche. However, in boys, pubertal development seems not to be linked to protein intake [39]. This gender gap can be explained by the fact that girls are intrinsically more prone to insulin resistance than boys [68]. Protein intake can also promote pubertal development mediated by the increasing IGF1 level [8]. Studies have reported that girls with higher IGF1 levels experienced earlier onset of thelarche and menarche [69,70]. Of note, the type of protein intake interferes with the association with circulating IGF1 concentrations [71], thereby having diverse impacts on puberty. Thus, further studies are warranted to shed light on the relationship between different types of protein intake and early puberty.

### 4.5. No Association between Early Puberty and Some Other Diets or Nutrients Intake

Controversy remains as to whether the total energy and carbohydrate intake affect the pubertal onset. We did not find the effects of energy and carbohydrate intake on early puberty in our study, but this finding was not robustly supported by sensitivity analysis. A previous meta-analysis suggested that girls with a higher level of total energy and carbohydrate intake were prone to earlier menarche onset [72]. Besides, a recent study found that the total energy intake was related to the timing of voice breaks in boys [39]. We infer that the real connection might be concealed by the study year, considering the marked change in the total energy intake in the past 30 years. Although we did not demonstrate the risk of earlier menarche for high fat intake, several possible mechanisms have been proposed. Leptin appears to link fat intake to the hypothalamic-pituitary-gonadal (HPG) axis. High fat intake can cause elevated leptin levels and even leptin resistance [73]. Leptin stimulates GnRH secretion via the regulation of Kisspeptin neurons [74], followed by accelerated puberty startup. High fat intake also initiates early puberty by inducing obesity [75,76]. Interestingly, high-fat diets can induce precocious puberty independent of obesity and leptin levels [77]. However, different types of fatty acids have divergent impacts on pubertal development directly [8] or through obesity [67], which deserves further research.

The impacts of micronutrients and phytochemicals on pubertal milestones remain poorly studied. Although a prior meta-analysis indicated a link between vitamin C and earlier menarche [72], evidence of the underlying mechanism is lacking. The intermediate impact of obesity may be a feasible research direction. However, much of the literature concerning the relationship between vitamins and obesity was controversial or contradictory [78,79]. Lignan, one kind of phytochemical, is defined as phytoestrogen due to its estrogenic and antiestrogenic properties [80,81]. As one kind of polyphenol [82], flavonols have a lot of effects against obesity via their antioxidant or anti-inflammatory properties [83]. However, we did not find their relationship with pubertal development.

Despite a positive relationship between milk consumption and IGF1 [84,85], we found that total milk intake was not correlated to menarche onset. The marginal result on low-fat milk and the uncorrelation result on total milk deserves attention. We boldly speculate that there may be a milk component associated with early puberty, but the milk fat renders this relationship indeterminate. With its similar structure to 17B-estradiol (E2), isoflavones interact with the HPG axis as phytoestrogens [86], affecting puberty progression [87]. As the main source of isoflavones, soy consumption had no effect on menarche time. This null finding can mainly be explained by the divergent soy intake habit and consumption in China and the USA.

### 4.6. Strengths and Limitations

Some strengths deserve to be highlighted. To date, this is the first meta-analysis to simultaneously investigate the association between diet or nutrient intakes during infancy and childhood and subsequent puberty timing. Second, the sample size of studies in our meta-analysis was relatively large. Ten studies were conducted in developing countries, and eighteen were done in developed countries. Since socioeconomic and demographic characteristics could be controlled to some extent, the findings were more generalizable. Third, the risk of early puberty was predominantly estimated with hazard ratios through prospective design. The above provided strong evidence for the causal relationship between early puberty and diet or nutrients. Furthermore, notwithstanding significant heterogeneity shown by several results, the quality of included studies was all moderate or high.

Some limitations also merit attention. We chose the risk estimates using the most-adjusted model, but the confounding factors adjusted in the included studies were still diverse. Besides, we selected the risk of early menarche in the highest intake group compared with the lowest or reference group, while the classification criteria of exposure groups were divergent in each research. Moreover, several methodologies were used to assess diet or nutrient intake, raising concerns about measurement accuracy in practice. Hence, the high heterogeneity would inevitably flaw the robustness of the final results. Unfortunately, we could not perform the subgroup analysis because of the scarcer studies. In addition, due to a lack of information to transform, the risk estimates (ORs, RRs, HRs) were directly described as the RRs. When the ORs are pooled with RRs and HRs, the risk of early menarche is overestimated.

## 5. Conclusions

Prolonged duration of breastfeeding, high intake of yogurt, and food insecurity had inverse associations with earlier menarche. In contrast, girls with a high intake of protein were more likely to experience earlier menarche. To sum up, the diet and nutrients in infancy and childhood may play a relevant role in pubertal development.

## Figures and Tables

**Table 1:** Characteristics of 28 included studies.

First Author	Year	Country	Study Design	Gender	Age (Years) ^a^	Sample Size	Diet or Nutrients	Diet/Nutrients Assessment	Puberty Outcome	Quality ^b^
Gaskins [27]	2017	Chile	C	F	3–4	389	dairy, milk, yogurt	DR	Age at menarche	8/9
Jansen [28]	2015	Colombia	C	F	8.4	456	red meat	FSQ	Age at menarche	7/9
Burris [29]	2020	USA	CS	F	13.2	40	food insecurity	FFQ	Age at menarche	7/11
Jansen [30]	2015	Colombia	CS	F	13.9	15,441	food insecurity		Age at menarche	8/11
Carwile [31]	2015	USA	C	F	9–14	5583	milk, low-fat milk, dairy, high-fat dairy, meat, red meat	FFQ	Age at menarche	7/9
Ramezani Tehrani [32]	2013	Iran	C	F	8.9	134	milk, yogurt	DR	Age at menarche	6/9
Belachew [33]	2011	Ethiopia	C	F	14.8	900	food insecurity	FSQ	Age at menarche	6/9
Rogers [12]	2015	UK	C	F	7	3298	total energy, protein, meat	FFQ/DR	Age at menarche	8/9
Koprowski [15]	1999	USA	C	F	10.6	1378	total energy, carbohydrate, protein, fat	FFQ	Age at menarche	7/9
Wiley [34]	2011	USA	C	F	9–12	3665	milk	DR	Age at menarche	6/9
Moisan [26]	1990	Canada	CC	F	10.7	666	total energy, protein, carbohydrate, fat, vitamin	DR	Age at menarche	7/9
Maclure [35]	1991	USA	C	F	10	194	total energy, protein, fat, vitamin	FFQ	Age at menarche	7/9
Meyer [36]	1990	Canada	C	F	10.9	109	total energy, carbohydrate, protein, fat	DR	Age at menarche	4/9
Xu [37]	2022	China	C	M/F	M:7.1 F:7	5920	fat	DR	Age at genital stage 2, breast stage 2, menarche, voice break	6/9
Koo [38]	2002	Canada	C	F	6–14	637	total energy	FFQ	Age at menarche	8/9
Meng [14]	2017	China	C	F	6–18	3199	total energy, carbohydrate, protein, fat	DR	Age at menarche	7/9
Cheng [39]	2022	UK	C	M/F	1 or 7	7730	total energy, carbohydrate, protein, fat	FFQ/FD	Age at menarche, genital stage 2, breast stage 2, voice break	8/9
Villamor [40]	2017	Colombia	C	F	8.6	1464	food insecurity	FSQ	Age at menarche	6/9
English [21]	2020	UK	C	M/F	1	4217	breastfeeding duration	Q	Age at menarche, voice break	8/9
Morris [22]	2010	UK	C	F	46	81,606	breastfeeding duration	Q	Age at menarche	5/9
Al-Mathkoori [23]	2018	Kuwait	CS	F	16.8	496	breastfeeding duration	TI	Age at menarche	7/11
Hvidt [24]	2021	Denmark	C	M/F	1	13,511	breastfeeding duration	TI	Age at genital stage 2,3,4,5; pubic hair stage 2,3,4,5; breast stage 2,3,4,5; menarche; voice break development	8/9
Segovia-Siapco [41]	2014	USA	CS	F	15	327	soy intake	FFQ	Age at menarche	8/11
Xiong [42]	2022	China	C	M/F	M:7.3 F:7.2	4781	soy intake	FFQ	Age at menarche, breast stage 2, gonadal growth stage 2, voice break	9/9
Kale [16]	2015	USA	C	F	6–8	1237	breastfeeding duration	Q/TI	Age at breast onset, age at pubic hair onset	8/9
Aghaee [25]	2019	USA	C	F	1	3331	breastfeeding duration	Q	Age at breast onset, age at pubic hair onset	9/9
Wolff [43]	2008	USA	CS	F	9.5	186	lignans, flavonols	FFQ	Age at breast, pubic hair development (stage = 1; 2+)	9/11
Mervish [44]	2013	USA	C	F	7.3	1178	lignans, flavonols	DR	Age at breast stage 2, pubic hair stage 2	8/9

Note: CS, cross-sectional study; C, cohort study; CC, case-control study; M, male; F, female; Q, questionnaire; FFQ, food frequency questionnaire; DR, dietary recall; FD, food diary; FSQ, food security questionnaire; TI, telephone interview. ^a^ Age (years): the age at baseline, mean or range. ^b^ Quality: The quality of cohort studies and case-control studies was evaluated with the Newcastle–Ottawa Scale (NOS). The quality of cross-sectional studies was assessed with the Agency for Healthcare Research and Quality (AHRQ) scale.

## Data Availability

Not applicable.

## Supplementary Materials

The following supporting information can be downloaded at: https://www.mdpi.com/article/10.3390/nu14235004/s1, Figure S1: Sensitivity analysis of studies for breastfeeding duration; Figure S2: Sensitivity analysis of studies for food insecurity; Figure S3: Sensitivity analysis of studies for (a) total energy intake, (b) carbohydrate intake, (c) protein intake, and (d) fat intake; Figure S4: Sensitivity analysis of studies for total milk intake; Table S1: Characteristics of 58 excluded studies; Table S2: Newcastle–Ottawa Scale for quality assessment of cohort studies and case-control studies.
