# Supplementary material for: Diet and Nutrients Intakes during Infancy and Childhood in Relation to Early Puberty: A Systematic Review and Meta-Analysis
Source: Nutrients. 2022 Nov 24;14(23):5004. doi: 10.3390/nu14235004 (PMC9739867; doi:10.3390/nu14235004)
Supplement: Supplementary file 1 [file nutrients-14-05004-s001.zip › nutrients-2006894-supplementary.pdf]

Supplementary materials

Supplemental Figure S1 Sensitivity analysis of studies for breastfeeding duration

Supplemental Figure S2 Sensitivity analysis of studies for food insecurity

Supplemental Figure S3 Sensitivity analysis of studies for (a) total energy intake, (b) carbohydrate intake, (c) protein intake, and (d) fat intake

Supplemental Figure S4 Sensitivity analysis of studies for total milk intake

Supplementary Table S1. Characteristics of 58 excluded studies

Supplementary Table S2. Newcastle–Ottawa Scale for quality assessment of cohort studies and case-control studies

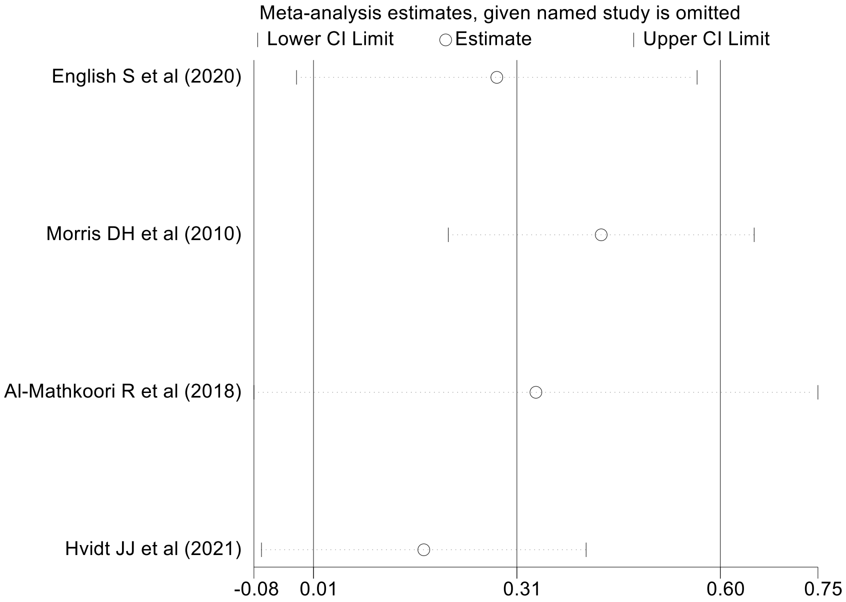

Supplemental Figure S1 Sensitivity analysis of studies for breastfeeding duration

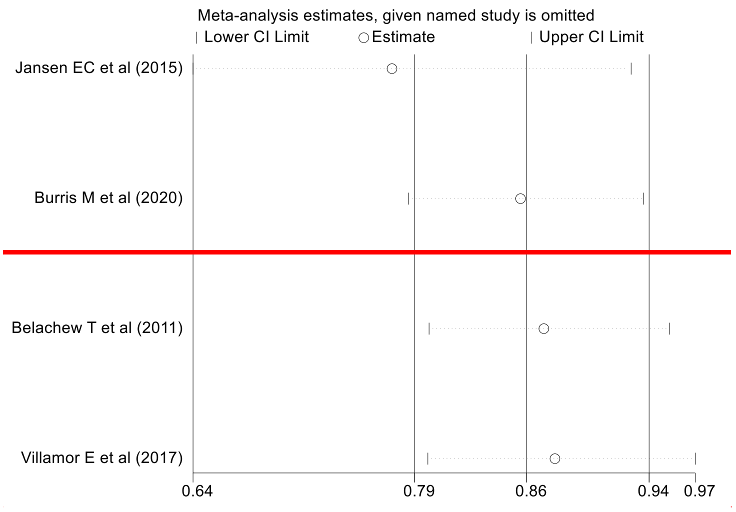

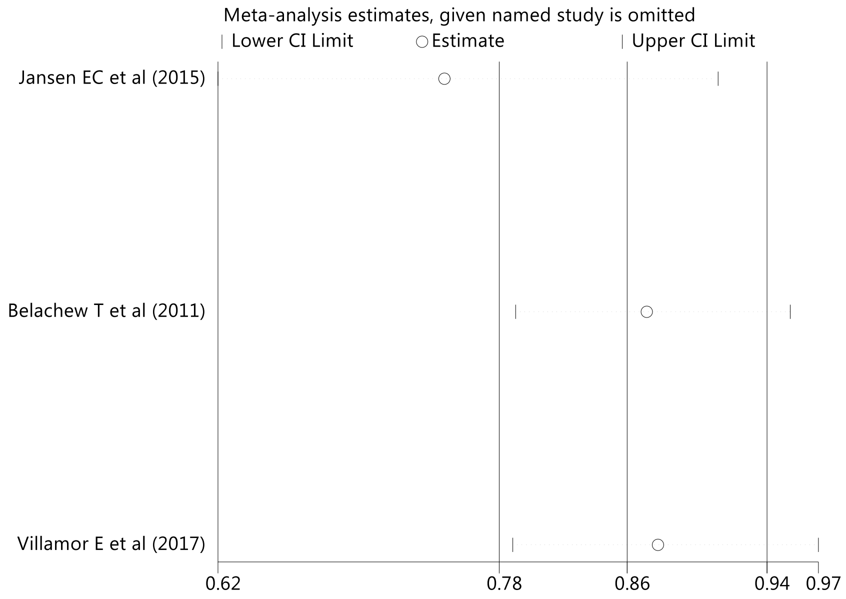

Supplemental Figure S2 Sensitivity analysis of studies for food insecurity

Given that food insecurity definitions differ in developed and developing countries, we ruled out the Burris et al study from the meta-analysis.

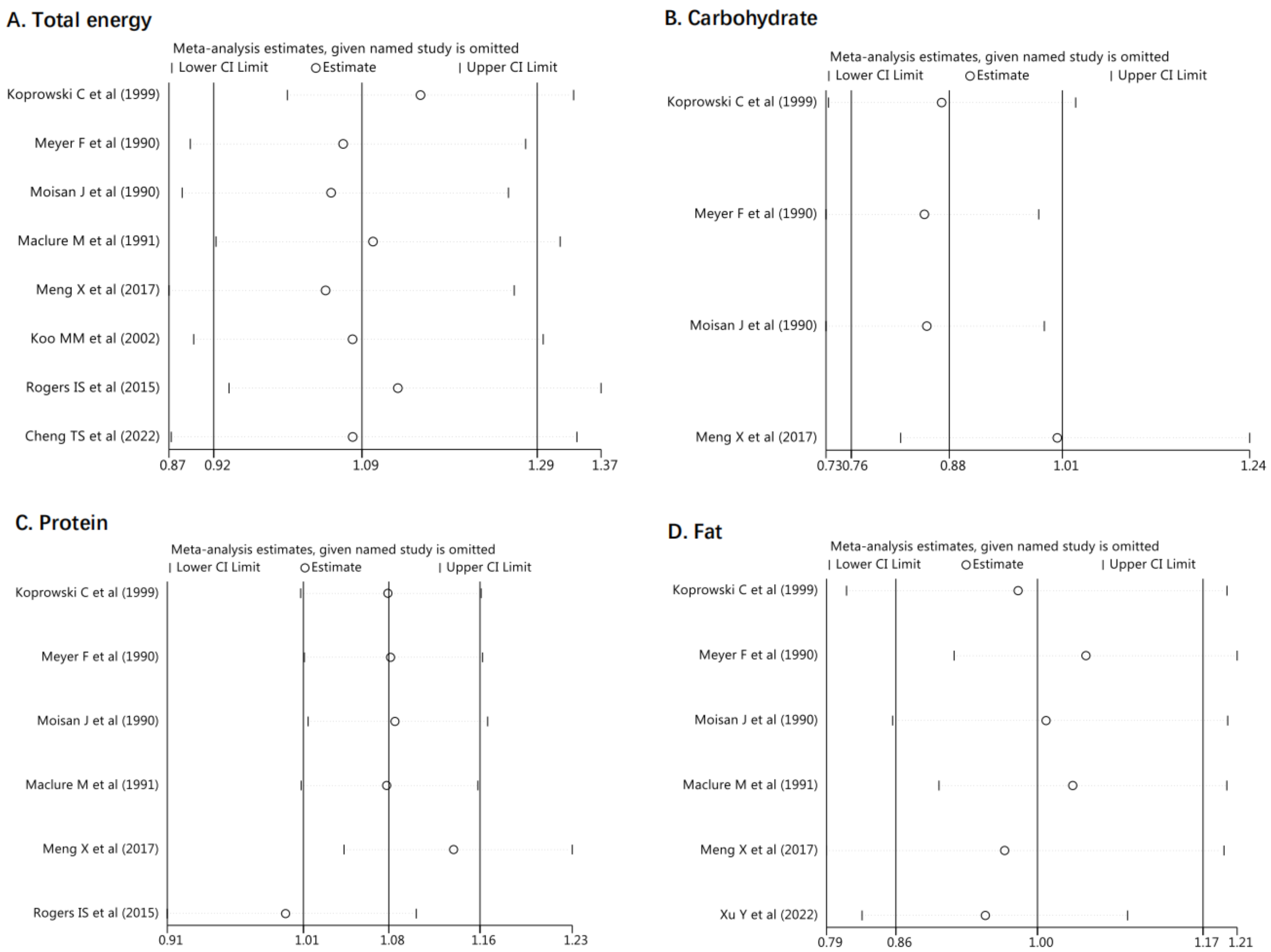

Supplemental Figure S3 Sensitivity analysis of studies for (a) total energy intake, (b) carbohydrate intake, (c) protein intake, and (d) fat intake

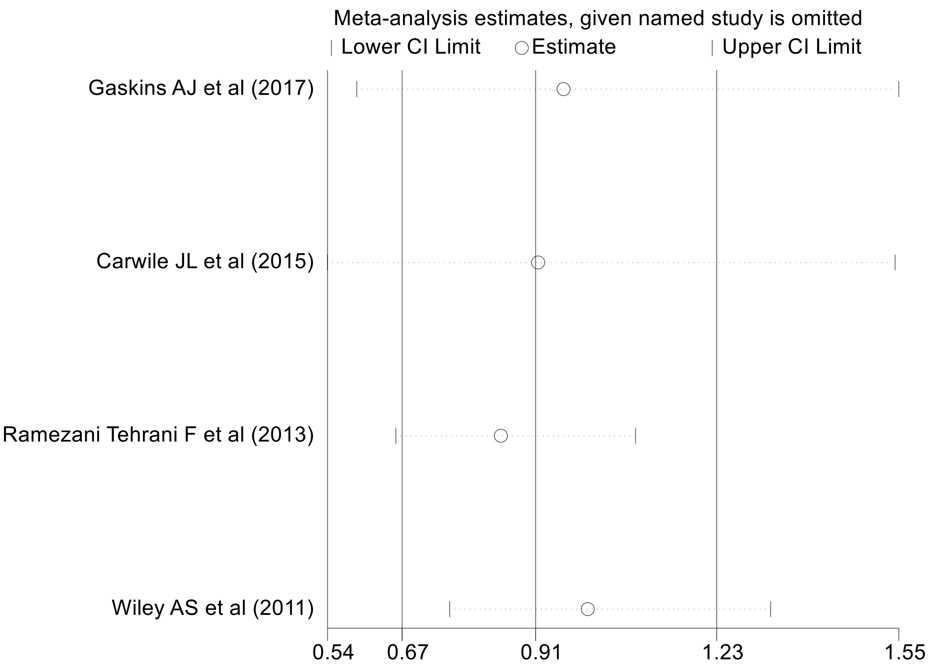

Supplemental Figure S4 Sensitivity analysis of studies for total milk intake

Supplementary Table S1. Characteristics of 58 excluded studies

No.	Study	Year	Country	Study design	Population	Gender	Age (years)	Sample size	Diet or food or Nutrients data	Pubertal development outcomes	Reasons for exclusion[a]
1	Bayrak	2020	Turkey	CS	135 healthy and 228 with celiac disease	M/F	healthy:12.92±2.35;CD: 12.77±2.25	363	Not mentioned	Age at menarche; Pubertal development (Tanner stages 1-5)	I
2	Garnier	2005	USA	C	Healthy	F	B:11.4±0.6	406	Not mentioned	Breast development stages; menarche	I
3	Abdou	2019	Lebanon	CS	Healthy	F	> 40	6032	Not mentioned	Age at menarche	I
4	Patel	2014	Canada	CS	29 healthy and 9 obese	M/F	8-18	38	Not mentioned	Pre-to-early puberty (Tanner stage 1, 2); Mid-to-late puberty (Tanner stage 3,4,5)	I
5	Buyken	2012	Germany	C	Healthy	M/F	B: 3m	1394	Not mentioned	Age at menarche	I
6	Atay	2011	Turkey	CS	Healthy	F	6-18	4868	Not mentioned	Puberty breast stages, puberty pubic-hair stages, menarche	I
7	Hashemipour	2018	Iran	CC	63 healthy and 87 with precocious puberty	F	case:8.12±1.2 control:8.31 ± 1.7	150	Not mentioned	Precocious puberty	I
8	Duan	2021	China	C	Healthy	M/F	B: M:7.1; F:7.3	3983	Diet quality according to CCDI score	Age at Tanner stage 2 for breast/genital development, menarche or voice break	I
9	Jansen	2018	Mexico	C	Healthy	M/F	B: 0 E: 14.5 ±2.1	496	Dietary pattern score	Age at manaeche, breast, genital, pubic hair stage≥4, testicular volume >15 ml	I
10	Dorgan	2003	USA	RCT	Healthy	F	B: 9.1±0.7	301	Dietary intervention group(fat intake<28%calories, cholesterol intake<150mg/d……)	Age at menarche	I
11	Cheng	2009	Germany	C	Healthy	M/F	B: 7.4± 1.3	222	Diet quality according to RC-DQI	Age at ATO	I
12	Chen	2018	China	CS	Healthy	M/F	6-12	6254	Traditional, unhealthy and protein diet	Precicious puberty	I

									pattern		
13	Sadov	2019	Finland	RCT	Healthy	M/F	B:7m; E:19y	193	Intervention group: low-saturated fat and low cholesterol diet	Tanner >G1P1; Tanner > B1P1	I
14	Belli	1988	Canada	RCT	With Crohn's disease and growth failure	M/F	intervention: 13.4±0.5 control:15.0 ± 0.7	12	Elemental diet, have no specific data	Pubertal development assessed by Tanner score	I
15	Szamreta	2020	USA	C	Healthy	F	B:10.02±0.58	202	Mediterranean-like Diet	Age at thelarche, menarche	I
16	Alimujiang	2018	USA	C	Healthy	M	B: 0; E: 18	64	Total fat, animal protein,vegetable protein	Not mentioned	II
17	Dorgan	2006	USA	RCT	Healthy	M	B:8-10	362	Total fat, saturated fat, dietary fiber	Not mentioned	II
18	Talpade	2001	USA	CS	Healthy	F	girl:6.2; women:76.3	48	Foods easten for breakfast,lunch, dinner without specific data	Not mentioned	II
19	Tassinari	2015	Italy	CS	With ICPP	F	8	31	Median PBDE serum levels	Not mentioned	II
20	Meng	2020	China	CC	Healthy;with precocious puberty	F	8	568	Dietary pattern: balanced, high calorie and high fat, high protein	Not mentioned	II
21	Guth	2021	Canada	CS	Healthy	F	11	382	Not mentioned	Not mentioned	II
22	Sekiyama	2015	Japan	C	Healthy	M/F	B: 0-12	418	Total energy, protein	Not mentioned	II
23	Bartkowicz	2018	Poland	CS	Healthy	M/F	M:17.6±1.1; F:17.4± 0.8	198	White bread, whole grain bread, grains and rice, dumplings and pasta, fruits, vegetables, milk, portk, yoghurts, cheese, white cheese, beef, poultry, meat products, fish, eggs, potato crisps,sweets/chocolate /bars, waffles, cookies, fast-food/hamburgers, hot-dogs, sodas, cola-like drinks, ice-cream	Not mentioned	II
24	Aurino	2017	UK	C	Healthy	M/F	B:younger:5, older:12	2891	Dietary diversity,cereals,roots, legumes,milk, eggs,fish,oil,fruit, vegetables	Not mentioned	II

25	Temple	2015	USA	RCT	Healthy	M/F	prepubertal: 8-9;postpubertal:15-17	112	Consume a 300 mL portion of a beverage containing either placebo or caffeine (1.0 or 2.0 mg/kg)	Not mentioned	II
26	Harris	2015	Germany	C	Healthy	M/F	B:10<br>E:15	1232	Total energy, protein , fat , carbohydrate, vegetables, fruit,meat, fish, starchy vegetables, whole grains, oils, refined grains, eggs, nuts and seeds, butter, margarine, dairy, sugar-sweetened foods, caloric drinks,PUFA,tea, water, retinol, Beta Carotene,Vitamin C, alpha tocopherol	Pre-puberty, early puberty, mid-puberty, late puberty, post-pubertal	II
27	de Castro	1998	USA	CS	Healthy	F	9.7-31.6	86	Carbohydrate, fat, protein	Not mentioned	II
28	Tukvadze	2015	Tbilisi	CS	Healthy	M/F	11-13	36	Total calories,protein, carbohydrate, mineral, Vitamin	Not mentioned	II
29	Talpade	2006	USA	CS	Healthy	F	8.13±1.1	45	Calcium, lipids	Early sexual maturation (any of breast, pubic hair development, hip enlargement)	II
30	Amador	1990	Cuba	RCT	Obese	M/F	B:10.6-12.9	78	Energy intake in the diet	Total ratings of sexual maturity: the Tanner score of genital+ pubic hair; the Tanner score of breast+ pubic hair	II
31	Mills	1986	USA	C	Healthy	M	B: 0;<br>E:14	78	Protein, calcium, iron, vitamin A, vitamin C, thiamine, riboflavin	Testlcular stretched volume 4.5-10, 11-14.9, 15-20, 21-25(cm3); Pubic hair stage 1-2,3,4,5; Tretched penlle length < 7.5, 7.5-9.9, 10-11.5, 12-14	III
32	Hill	1980	USA	CS	Healthy	F	8-14	242	Total calorie intake daily, carbohydrates, fat, animal protein, vegetable protein	Age of menarche	III
33	Niinikoski	2007	Finland	RCT	Healthy	M/F	B: 7 m ;E: 14	1062	Total energy, fat, saturated fat,protein,carbohydrate	Pubertal Stage 1-5; Ages at menarche	III
34	Alemu	2021	Ethiopia	CS	Healthy	F	14.7±2.29	757	Dietary diversity, food security	Age at first menstruation	III

35	Shahatah	2021	Saudi	CS	Healthy	F	11.7±2.8	164	Fast food consumption; Organic poultry	Age at thelarche; Age at menarche	III
36	Mueller	2015	USA	C	Healthy	F	B:9-10	1988	Soft drinks, natural fruit juices; Caffeine, Aspartame, Sucrose, Fructose	Early menarche (<11y)	IV
37	Burris	2021	USA	C	Healthy	F	E: 12-15	424	Food security	Age at menarche	IV
38	Wu	2021	China	CS	Healthy	F	6-18	1981	Pork,beef,lamb,poultry, processed meat, total meat	Age at menarche	
39	Günther	2010	Germany	C	Healthy	M/F	E:6-13	112	Total protein,animal protein,vegetable protein	Age at take-off of the pubertal growth spurt; age at peak height velocity	IV
40	Patel	2016	Canada	RCT	Healthy	M/F	9-14	36	Food intake, water intake	Pre-early pubertal and mid-late pubertal	IV
41	Simon	2003	UK	C	Healthy	M/F	B: F:11.85±0.32;M:11.81±0.37	4320	Fruit and vegetable, high-fat food intake: score from 0-10	Pre-, beginning, mid-, advanced, post puberty	IV
42	Xie	2021	China	RCT	Obese	F	6-10	62	Decaffeinated green tea polyphenols	Pubic hairs, ampits, breast development	IV
43	Luo	2016	China	CS	Healthy	M/F	male:9.05 female:9.20	1425	Fried and puffed food, out-of-season vegetables	Age at testicular volume (4-12ml); menarche, PH2, B2; first spermatorrhea	IV
44	Van Jaarsveld	2007	UK	CS	Healthy	M/F	11-12	5229	High-fat diet ; daily breakfast routine	Early, average and late puberty	IV
45	Schraw	2015	Norway	C	180 normotensive and 108 preeclamptic	F	B: 0 E:12.9	228	Breastfeeding duration, formula,solids	Breast development (stage 1-5)	IV
46	Lauritzen	2016	Denmark	RCT	Healthy	M/F	B: 0 E: 13	100	4·5 g/d Fish oil (with 1·5 g/d n-3 LCPUFA) or 4·5 g/d Olive oil	F: breast development; M: pubic hair developemt	IV
47	Britton	2004	USA	CS	Healthy	F	9	186	Calories, protein, carbohydrate,fat,crude fiber, Vitamin	Prepubertal (stage 1) or pubertal (stages 2–5) for breast and pubic hair development	IV
48	Clavien	1996	Switzerland	CS	Healthy	M/F	9-19	193	Lipids, proteins, carbohydrates	Puberty stage 1-5	IV

49	Cheng	2019	Australia	C	Healthy	M/F	B: 8	142	Total energy, protein, carbohydrate, fat	F: age at menarche, Tanner B3<br>M: age at Tanner G3	IV
50	Chen	2014	China	CC	With ICPP	F	6.88±0.04	1113	Fruit,vegetable,poultry meat,instant noodles	Idiopathic central precocious puberty	IV
51	Vannucci	2014	USA	RCS	Healthy	M/F	8-17	468	Carbohydrate, fat, protein in people with and without loss of control eating	Prepuberty, early-midpuberty, late puberty	IV
52	Shomaker	2010	USA	CS	Healthy	M/F	8-17	204	Total energy,carbohydrate, fat, protein	Prepuberty, early-midpuberty, late puberty	IV
53	Xu	2019	China	CC	Healthy	F	Case: 5.3 ± 0.7; Control: 5.5 ± 0.8	485	Total energy,carbohydrate,protein,fat intake	Premature thelarche(Tanner stage 2 and 3)	IV
54	Karaolis-Danckert	2009	Germany	C	Healthy	M/F	B:0	215	Breastfeeding duration	Age at peak height velocity	IV
55	Blell	2008	UK	C	Healthy	F	50	276	Breastfeeding duration	Early menarche, average menarche, late menarche	IV
56	Lee	2015	Korea	C	Healthy, precocious	M/F	7-9	219	Breastfeeding duration	Early pubertal development (Tanner stage2+)	IV
57	Kwok	2012	China	C	Healthy	M/F	B:0	7523	Breastfeeding duration,milk consumption (at about 6 months, 3 years, and 5 years)	Age at pubertal onset (Tanner stage 2)	IV
58	Al-Sahab	2011	Canada	C	Healthy	F	B:0	994	Breastfeeding duration	Age at Menarche	IV

Note: B, the age at baseline; E, the age at the end of the study; CS, cross-sectional study; C, cohort study; CC, case-control study; RCT, randomized controlled trial; RCS, randomized crossover study; M, males; F, females. [a]The reasons for exclusion: I, no exact data on diet or food or nutrients. II, no puberty outcome or not judged according to Tanner staging method. III, no quantized data showing the relationship between puberty outcome and diet or food or nutrients. IV, the one article to show the relationship and cannot be combined using meta-analysis.

Supplementary Table S2. Newcastle–Ottawa Scale for quality assessment of cohort studies and case-control studies

Study	Selection	Comparability	Outcome or Exposure	Total score	Quality[a]
Cheng [27]	★★★★	★★	★★★	9	high
Villamor [82]	★★★★		★★	6	moderate
English [21]	★★★★	★★	★★	8	high
Morris [22]	★★★		★★	5	moderate
Gaskins [72]	★★★★	★★	★★	8	high
Jansen [73]	★★★	★★	★★	7	high
Carwile [75]	★★★	★★	★★	7	high
Ramezani Tehrani [76]	★★★	★★	★	6	moderate
Belachew [77]	★★★	★★	★	6	moderate
Koprowski [15]	★★★	★★	★★	7	high
Wiley [78]	★★★	★★	★	6	moderate
Maclure [79]	★★★	★★	★★	7	high
Meyer [30]	★★		★★	4	moderate
Meng [14]	★★★	★★	★★	7	high
Xu [80]	★★	★★	★★	6	moderate
Koo [81]	★★★★	★★	★★	8	high
Rogers [12]	★★★★	★★	★★	8	high
Hvidt [24]	★★★★	★★	★★	8	high
Moisan [26]	★★★★		★★★	7	high
Xiong [84]	★★★★	★★	★★★	9	high
Kale [16]	★★★	★★	★★★	8	high
Aghaee [25]	★★★★	★★	★★★	9	high
Mervish [29]	★★★	★★	★★★	8	high

Note: The cohort studies were evaluated from three aspects of selection,comparability and outcome.The case-control studies were evaluated from three aspects of selection,comparability and exposure. A star is given if the study meets one item of NOS and the highest score is 9. [a]Quality: low, 0-3 scores; moderate, 4-6 scores; high, 7-9 scores.
